# *GmWRKY45* Enhances Tolerance to Phosphate Starvation and Salt Stress, and Changes Fertility in Transgenic *Arabidopsis*

**DOI:** 10.3389/fpls.2019.01714

**Published:** 2020-01-29

**Authors:** Cheng Li, Xinyi Liu, Hui Ruan, Jingyao Zhang, Fengbin Xie, Junyi Gai, Shouping Yang

**Affiliations:** Soybean Research Institute, National Center for Soybean Improvement, Key Laboratory of Biology and Genetic Improvement of Soybean (General, Ministry of Agriculture), State Key Laboratory of Crop Genetics and Germplasm Enhancement, Jiangsu Collaborative Innovation Center for Modern Crop Production, Nanjing Agricultural University, Nanjing, China

**Keywords:** *GmWRKY45*, soybean (*Glycine max*), transgenic *Arabidopsis*, tolerance, phosphate starvation, salt stress, fertility

## Abstract

WRKY protein is a unique transcription factor (TF) and plays an important role in the physiological processes of various stress responses and plant development. In this research, we obtained a WRKY TF gene from soybean by homologous cloning, and named it *GmWRKY45*. *GmWRKY45* is a nuclear protein containing a highly conserved WRKY domain and a C_2_H_2_ zinc finger structure, and mainly expressed in roots, flowers and pods of soybean. The quantitative reverse transcription–PCR showed that *GmWRKY45* was induced by phosphate starvation and salt stress. As compared with the wild type (WT), overexpression of *GmWRKY45* increased the adaptability of transgenic *Arabidopsis* to phosphate starvation, which might be related to the enhancement of lateral root development. The phosphorus concentration, fresh weight and dry weight of *GmWRKY45*-overexpressing *Arabidopsis* were higher than those of WT under Pi-sufficient or Pi-deficient condition. Meantime, the expression of phosphate-responsive genes was affected in transgenic *Arabidopsis.* Furthermore, *GmWRKY45* improved the salt tolerance and changed fertility of transgenic *Arabidopsis*. Under salt stress, we found the survival rate and soluble sugar content of transgenic *Arabidopsis* were significantly higher than those of WT. In a conventional soil pot experiment, the transgenic *Arabidopsis* produced shorter silique, less and larger seeds than WT, these might be due to partial abortion of pollens. The overall results showed that *GmWRKY45* was not only involved in response to abiotic stress but also related to fertility, suggested that *GmWRKY45* had an elaborate regulatory system in plants.

## Introduction

In the natural environment, plants will encounter various biological and non-biological pressures during their growth and development. Phosphorus (P) is one of 17 essential elements required for plant growth. P plays an important role in plant growth and development, including energy generation, nucleic acid synthesis, photosynthesis, glycolysis, respiration, membrane synthesis and stability, etc. Although P is abundant in many soils, crop yield on 30%–40% of the world’s arable land is limited by P availability, because plants take up P exclusively in the form of inorganic phosphate (Pi) (Vance et al., 2003). Soil salinity is also a major abiotic stress worldwide (Rengasamy, 2006). In irrigated land areas, continuous salinization of agricultural land is a serious threat to crop yields (Munns and Tester, 2008). Therefore, the development of plants that can efficiently resist phosphate starvation and salt stress are a sustainable and economic approach for plant production, thus it may be an important contribution for the improvement of crop yield.

In recent years, scientists have discovered several key molecules involved in Pi signal transduction and salinity tolerance, further improving the understanding to plant phosphorus utilization and salt tolerance at the molecular level (Zhang et al., 2016; Xu et al., 2018). By using genetic methods, many families of transcription factors have been found to play important roles in plant phosphorus signaling and salt tolerance, including WRKY, AP2/ERF, MYB, bHLH, and NAC family members (Baek et al., 2013; Phukan et al., 2016). These TFs usually bind to specific cis-acting elements in the promoter region and regulate the expression of several stress response genes. For example, AP2/ERF can be combined with GCC box and DRE/CRT components and induced the expression of some PR genes, thus improving the high salt tolerance of plants (Zhang et al., 2009).

The WRKY TF superfamily is one of the largest TF families and almost all are found in plants. The WRKY transcriptional factor superfamily has 72 members in *Arabidopsis* (*Arabidopsis thaliana*) and 182 members in soybean (*Glycine max*), and its definition is based on the highly conserved WRKY domain (Eulgem and Somssich, 2007; Bencke-Malato et al., 2014). The WRKY domain is a conserved DNA-binding region that includes highly conserved WRKYGQK peptide sequences and zinc finger motifs which can be either C_2_H_2_-type (Cx_4-5_Cx_22-23_HxH) or C_2_HC-type (Cx_7_Cx_23_H_x_C). Members of the WRKY family have been found to contain at least one such domain. This domain generally binds to the promoter region of target genes containing the W-box sequence (C/TTGACT/C), although alternative binding sites have been identified (Pandey and Somssich, 2009). W-box elements are prevalent in plant genomes. For example, 32,162 TTGACY, 60,612 TTGAC, and 14,857 TTTGACY were identified in *Arabidopsis* (Chen et al., 2017). The current and widely accepted system of WRKY classification was established in 2000 based on the genomic characterization of this gene family in *Arabidopsis* (Eulgem et al., 2000). All WRKY TFs are classified into three categories: I, II, and III, based on the number of WRKY domains and the type of zinc finger figure. Group I WRKY proteins harbor two WRKY domains and a C_2_H_2_-type zinc-finger structure, whereas groups II and III WRKY proteins contain only one WRKY domain with C_2_H_2_ and C_2_HC type zinc-finger structures, respectively. Group II proteins are further categorized into five subgroups (IIa–IIe) (Rushton et al., 2010). WRKY transcription factors have been shown to be involved in responses to biotic and abiotic stresses, and in developmental processes (Eulgem and Somssich, 2007). Transgenic *Arabidopsis* plants over-expressing *GmWRKY13* showed increased sensitivity to salt and mannitol stresses, and decreased sensitivity to abscisic acid (ABA), when compared with wild-type plants (Zhou et al., 2008). *GmWRKY21*-transgenic *Arabidopsis* plants were tolerant to cold stress (Zhou et al., 2008). *GmWRKY54*-transgenic *Arabidopsis* plants conferred salt and drought tolerance (Zhou et al., 2008). According to the newest publication, *AtWRKY45* overexpression in *Arabidopsis* increased Pi content and uptake, while RNA interference suppression of *AtWRKY45* decreased Pi content and uptake (Wang et al., 2014). Since *Arabidopsis* is a dicotyledonous model plant, we speculate that *GmWRKY45* in soybean may have a similar function. However, WRKY protein with structural homology in different plants may have different functions, which needs experimental verification (Yang et al., 2016).

Soybean is one of the most important crops for oil and protein production, and its yield is severely affected by various environmental conditions (Keaton et al., 2013). In this research, we cloned the full-length cDNA of *GmWRKY45*, and report its expression under phosphate starvation and salt stress. We found that overexpression of *GmWRKY45* increased tolerance to phosphate starvation and salt stress in transgenic *Arabidopsis*. Interesting, we also found the transgenic *Arabidopsis* produced shorter silique, less and larger seeds. These results showed that *GmWRKY45* was not only involved in response to abiotic stress, but also related to fertility, suggesting that *GmWRKY45* had an elaborate regulatory system in plants.

## Materials and Methods

### Phylogenetic and Gene Structure Analysis

Using *AtWRKY45* cDNA sequence of *Arabidopsis* as probe, BLAST retrieval was conducted using the National Center of Biotechnology Information (www.ncbi.nlm.nih.gov/), find the target gene. BioXM 2.6 software was used to predict the molecular weight and isoelectric point of the gene, and GSDS (http://gsds.cbi.pku.edu.cn/) online program was used to predict the gene structure. The conserved functional domains of the gene were predicted by using SMART (http://smart.embl-heidelberg.de/) and NCBI conserved structural domain database (CDD) (https://www.ncbi.nlm.nih.gov/Structure/cdd/wrpsb.cgi). ClustalX 1.83 and GeneDoc were used for multiple alignments. Neighbor-joining phylogenetic trees were generated using the MEGA 5.1 program.

### Plant Material, Growth Conditions, and Stress Treatments

Seeds of soybean genotype Williams 82 were used to investigate the tissue expression pattern of *GmWRKY45* and its responses to various stresses including phosphate starvation and salt stress. Phosphate starvation: Soybean seeds germinated in vermiculite medium for five days were transferred to Hoagland solution and treated with two Pi levels (low Pi, 2.5 µM KH_2_PO_4_; normal Pi, 1 mM KH_2_PO_4_) for 10 days (removal of cotyledons). A low Pi group re-supplied with Pi for 1 day was called Recovery 11d. Salt stress: Soybean seeds germinated in vermiculite medium for five days were transferred to Hoagland solution and treated with two NaCl levels (0 mM NaCl; 150 mM NaCl) for five days (removal of cotyledons). A 150 mM level NaCl group was transferred to 0 mM NaCl medium for 1 day, called Recovery 6d. Leaves and roots were sampled after transfer. All samples were frozen in liquid nitrogen and stored at −80°C prior to RNA extraction.

### Quantitative Real-Time PCR (RT–qPCR)

Three independent biological replicates, each comprising three individual plant, were used for quantitative real-time PCR. The genomic DNA was removed and the total RNA was converted to cDNA using the HiScript II 1st Strand cDNA Synthesis Kit (Vazyme Biotech, Nanjing, China). Quantitative real-time PCR was performed on a BioRad CFX96 real time system and the products were labeled using SYBR qPCR Master Mix (Vazyme Biotech, Nanjing, China). Triplicate quantitative assays were performed on each cDNA sample. All primers used for RT-qPCR are listed in **Supplemental Tables S1** and **S2**.

### Arabidopsis Protoplast Isolation and Subcellular Localization

The coding sequence (CDS) without a termination codon of *GmWRKY45* (861 bp) was used to construct the *pJIT166-GmWRKY45-GFP* vector driven by the *CaMV35S* promoter. *Arabidopsis* protoplasts were isolated by following the previously published protocol (Yoo et al., 2007). Both fusion (*pJIT166-GmWRKY45-GFP*) and control (*pJIT166-GFP*) constructs were transformed into *Arabidopsis* protoplast by the PEG4000-mediated method as described by Abel and Theologis (1994). The GFP fluorescence was imaged using a Zeiss LSM780 camera (Carl Zeiss, SAS, Jena, Germany).

### Development of Transgenic Arabidopsis

To generate *GmWRKY45*-overexpressing transgenic *Arabidopsis* lines, full-length cDNA of the *GmWRKY45* gene from Williams 82 was amplified. An over-expression construct was generated by inserting a full-length *GmWRKY45* cDNA fragment into the binary vector *pCAMBIA3301-GFP* after the *CaMV35S* promoter, using the Gateway Technology with a One Step Cloning Kit (Vazyme Biotech, Nanjing, China) according to the manufacturer’s protocol. The sequences of the primers are provided with **Supplemental Table S3**.

In this study, *Arabidopsis* ecotype Columbia-0 (Col-0) was used. The *pCAMBIA3301-GmWRKY45* vector was introduced into *Agrobacterium tumefaciens* strain EHA105 *via* the freeze-thaw method. The *Agrobacterium*-mediated floral dip method was used for *Arabidopsis* transformation (Clough and Bent, 1998). Transgenic plants were selected on Basta (20 mgL^−1^) medium, and the selected plants were identified by PCR (**Supplemental Figure S1**). Homozygous T3 or T4 seeds were used for researching.

### Measurement of Chlorophyll and Anthocyanin Contents

Two-week-old plants were grown in Hoagland medium with two Pi levels (P+, 1mM KH_2_PO_4_; P-, 0.25mM KH_2_PO_4_) for 30 days. The chlorophyll content was assayed by SPAD-502 Plus (Japan). The anthocyanin content was assayed referred to Kim et al. (2003) and a little modification: Take plant leaf blade 0.01 g, in 300 ul 1% HCl-methanol mixture, 4°C temperature overnight, then add 200 µl ddH_2_O_2_ and 200 ul trichloromethane, 14,000 rpm centrifuge for 15 min and drain the upper water phase into the enzyme label plate, enzyme standard instrument for determination of 530 nm and 657 nm absorbance value. Anthocyanin formula: A_530-_0.33A_657_. Three plant samples were randomly selected from each treatment, and each experiment contained three biological replicates.

### Measurements of Lateral Root Length and P Concentration

Seedlings were grown on square petri dishes under P+ (1 mM KH_2_PO_4_) and P- (0.25 mM KH_2_PO_4_) milieu for 15 days. The roots were photographed under the scanner (Epson, Expression 11000XL, Japan) The length of lateral roots in individual plants was measured using the ImageJ program (Abràmoff et al., 2004). Data were recorded from five individual plants from each line per treatment. Inorganic phosphorus and total phosphorus concentration were determined using Tissue Total Phosphorus Content Detection Kit (Solarbio, Beijing, China) and Tissue Inorganic Phosphorus Content Detection Kit (Solarbio, Beijing, China), respectively. Each experiment contained three biological replicates.

### Measurement of Survival Rate and Soluble Sugar Content

For survival assay, surface sterilization was carried out on 24 seeds of *GmWRKY45*-overexpressing transgenic *Arabidopsis* and WT, and they were placed in Petri plates containing Murashige and Skoog (MS) basal medium supplemented with 0 and 150 mM NaCl, respectively. The petri dishes were placed in a growth chamber at 25°C for 7 days under 16 h photoperiod. The survival rate was recorded as the ratio of plants with true leaf normal greening after 7 days to the total number sown.

Two-week-old plants were transferred MS nutrient solution supplemented or not with 150 mM NaCl and grown for 5 days. Soluble sugar contents were referring to a method of Xiang et al. (2007). 0.01 g *Arabidopsis* fresh leaf was cut into debris and put into a clean tube; 1ml of ddH_2_O was added and then the tube was placed in a boiling water bath for 30 min to extract. After cooling to room temperature, 0.2 ml extracts were taken and placed in another clean tube, added 1 ml of 5% phenol and 5 ml of concentrated sulphuric acid. The mixture was then well shaken. After standing for 30 min, the aqueous extract was assayed for soluble sugar content at the wavelength of 485 nm. Each experiment contained three biological replicates.

### Alexander Staining of Pollen and Measurement of Seed Size

Alexander stain is a kind of plant pollen dye commonly used for nuclear staining. Plant pollen using this stain will turn purplish red. Take fresh flowers, carefully remove the petals and pistil, put the pollen on a glass slide, and add 2–3 drops of Alexander Stain, mix them thoroughly, immediately cover the glass slide, remove the excess liquid and observe under the microscope.

The seed size was photographed under an OLYMPUS CX31 stereo microscope with a SPOT-RT digital camera attached to a computer (Olympus, Melville, NY, USA). The length and width of the seed was measured using the ImageJ program (Abràmoff et al., 2004). Data were recorded from three individual plants with 10 seeds used from each line.

### Promoter Cloning and GUS Assay

To study the promoter activity, a 2-kb genomic region upstream of the translation initiation codon of *GmWRKY45* gene was cloned into *pCAMBIA3301-GUS* at *BamH*I and *Xba*I sites (primers see **Supplemental Table S4**). The sequence correctness of the target promoter on the fusion construct was verified by sequencing. The fusion construct was introduced into *Agrobacterium tumefaciens* strain EHA105 by electroporation. The *Agrobacterium*-mediated floral dip method was used for *Arabidopsis* (Col-0) transformation (Clough and Bent, 1998).

GUS assay procedures were the same as described by Scarpeci et al. (2008).

### Statistical Analyses

Statistical analyses were performed using Microsoft Excel 2003 and The SAS System for Windows V8. Significant differences were evaluated using the two-tailed Student’s *t*-test or one-way ANOVA and Duncan’s test. All test differences at P ≤ 0.05 were considered to be significant. All the error bars were SD (Standard Deviation) value.

## Results

### Structure, Homology Characterization, and Subcellular Localization of GmWRKY45

**Figure 1:**
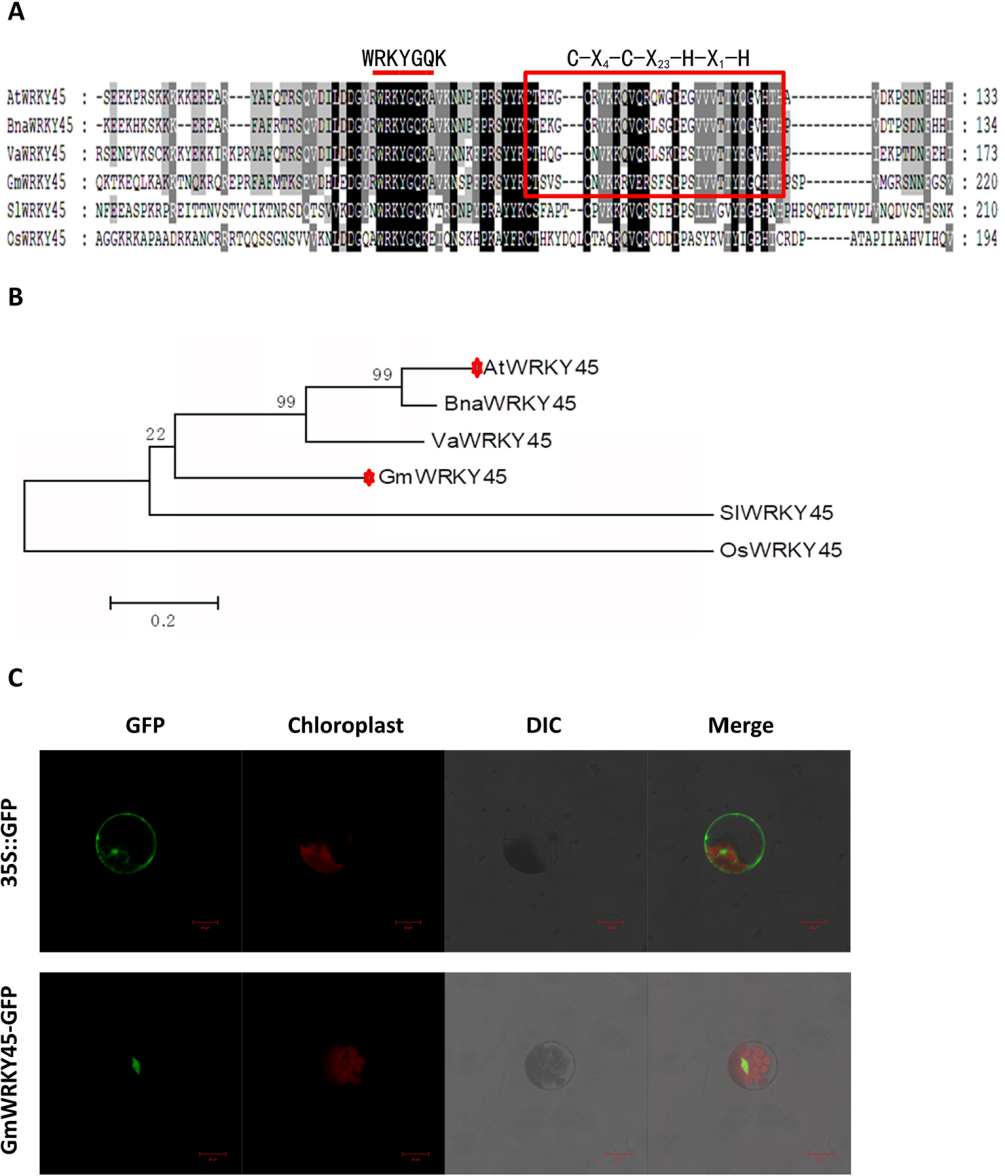
Structure, homology characterization and subcellular localization of *GmWRKY45*. **(A)** GmWRKY45 and the WRKY45 protein from *Arabidopsis* (*AtWRKY45*), *Oryza sativa* (*OsWRKY45*), *Vitis amurensis* (*VaWRKY45*), *Brassica napus* (*BnaWRKY45*), and *Solanum lycopersicum* (*SlWRKY45*) were compared and analyzed by ClustalX 1.83 software. The same amino acids in all six proteins are shown in dark gray. The red bar above the sequences represents the highly conserved WRKYGQK domain. Red square represents the N-terminal C-X_4_-C-X_23_-H-X_1_-H zinc finger structure. **(B)** Based on the amino acid sequence of the selected GmWRKY45 protein, a phylogenetic comparison was made between the sequences of the GmWRKY45 protein and the WRKY45-related protein. The unrooted tree was constructed using MEGA 5.1 by the neighbor-joining method with bootstrap probabilities based on 1000 replicates shown at branch nodes. Accession numbers for the WRKY45 proteins used are as follows: *GmWRKY45*, NP_001237422; *AtWRKY45*, NP_186846; *OsWRKY45*, AAW63720; *VaWRKY45*, AFK27601; *BnaWRKY45*, ACH99806, and *SlWRKY45*, NP_001304843. **(C)** Subcellular localization of GmWRKY45-GFP and 35S::GFP protein in *Arabidopsis* protoplasts. Scale bar = 20 µm.

According to the coding sequence (CDS) of *AtWRKY45* (AT3G01970) gene in *Arabidopsis thaliana*, through NCBI database and Phytozome 11.0 database, homologous gene comparison was conducted to find the gene sequence with the highest homology with *AtWRKY45* gene in soybean, which I’ll call *GmWRKY45* (Glyma.03G220800). *GmWRKY45* was located on chromosome 3 of soybean, position Gm3 (42405222 - 42407091). Sequence analysis showed that it contained 3 exons, an 864 bp open reading frame encoding a polypeptide of 288 amino acids with predicted molecular mass of 32.37 kDa (pI 6.95). The amino acid sequence encoded by the gene was compared analysis on NCBI website, and it was found that the protein encoded by *GmWRKY45* had a conservative WRKY domain sequence. The amino acid sequence of *GmWRKY45* (NP_001237422) was used as the probe, and the WRKY45 protein in *Arabidopsis*, *Oryza sativa*, *Vitis amurensis*, *Brassica napus* and *Solanum lycopersicum* was obtained by NCBI BLASTp tool: *AtWRKY45* (NP_186846), *OsWRKY45* (AAW63720), *VaWRKY45* (AFK27601), *BnaWRKY45* (ACH99806) and *SlWRKY45* (NP_001304843). Multiple comparisons showed that the amino acid sequence of *GmWRKY45* was relatively conservative, and the N-terminal of *GmWRKY45* protein contained a WRKYGQK domain and a C-X_4_-C-X_23_-H-X_1_-H zinc finger structure (**Figure 1A**), so *GmWRKY45* belonged to the IIc subfamily in the WRKY transcription factor family (Chen et al., 2017). Phylogenetic trees constructed from amino acid sequences showed high homology between *GmWRKY45* and *AtWRKY45* (**Figure 1B**), so we speculated that they may have similar functions.

Next, we predicted the subcellular localization of GmWRKY45 protein through the website (http://www.csbio.sjtu.edu.cn/bioinf/Cell-PLoc-2/), (Chou and Shen, 2008), and the predicted results showed that it was located in the nucleus. To further verify the subcellular localization of *GmWRKY45*, the full-length *GmWRKY45* cDNA without the termination codon was fused in-frame to the 5’ end of *GFP* gene of the *pJIT166* vector to obtain the pJIT166-*GmWRKY45* fusion construct. By following the PEG-4000-mediated method, the fusion plasmid of *pJIT166-GmWRKY45* and the *pJIT166* vector as control were introduced into *Arabidopsis* protoplasts. The cells were observed by confocal laser scanning microscope. The fluorescence of GmWRKY45-GFP accumulated mainly in the nucleus, while the fluorescence of the control was distributed evenly throughout all parts of the cell including the nucleus and cytoplasm (**Figure 1C**). These results indicate that GmWRKY45 likely functions in the nucleus and has the general characteristics of transcription factors.

### Expression Patterns of GmWRKY45

**Figure 2:**
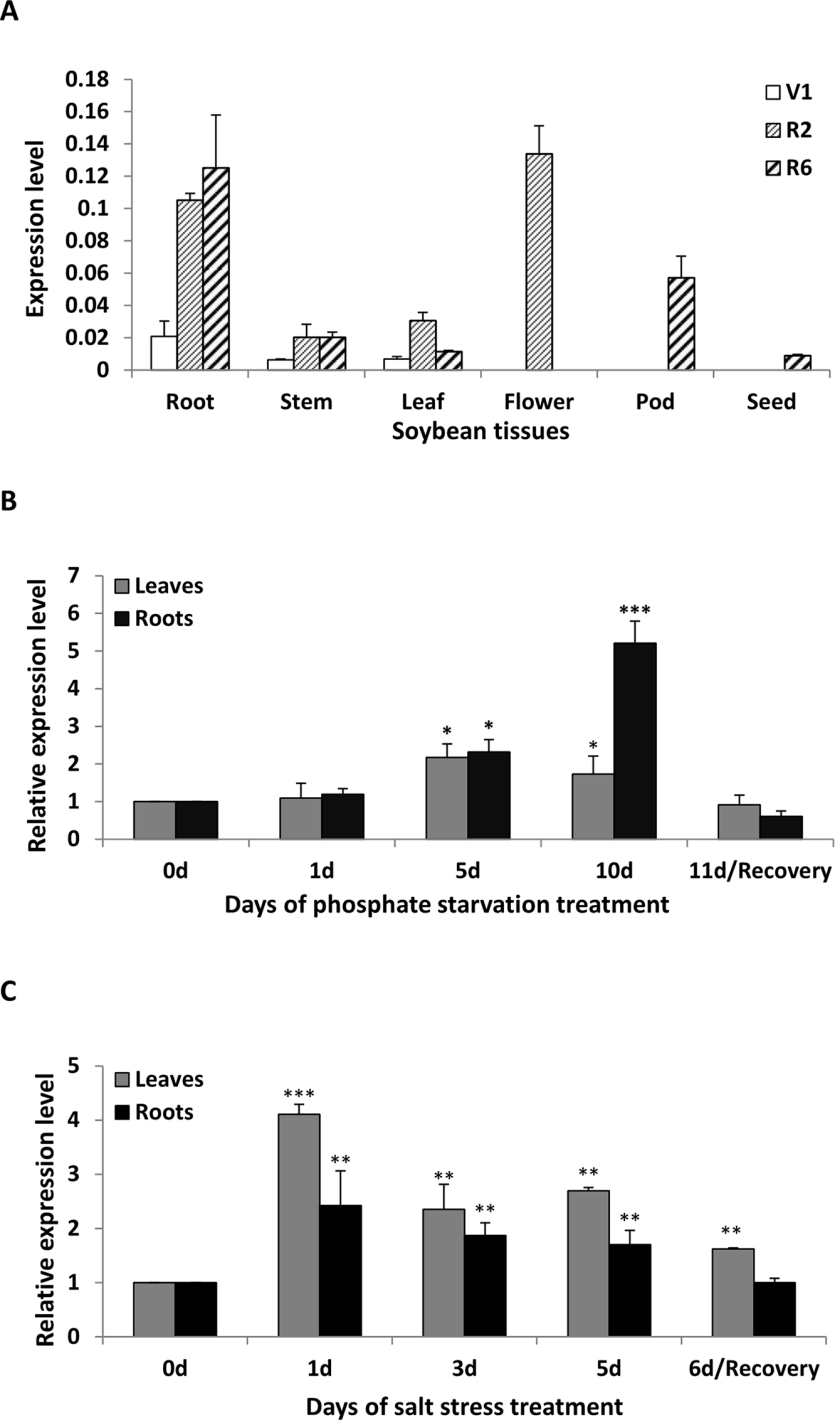
Expression patterns of *GmWRKY45* in soybean. **(A)** The expression of *GmWRKY45* in roots, stems, leaves, flowers, pods and seeds at the first trifoliate stage (V1), full bloom stage (R2) and full seed stage (R6). **(B)** Time course of the expression level of *GmWRKY45* in leaves and roots. Expression is relative to the transcript levels under Pi-sufficient condition (2.5 µM Pi). 0, 1, 5 and 10 d, duration of Pi starvation (days); Recovery 11 d, 10 days of Pi starvation followed by 1 day on Pi-sufficient substrate. **(C)** Time course of the expression level of *GmWRKY45* in response to salt stress (150 Mm NaCl). 0, 1, 3, and 5 d, duration of salt stress (days); recovery 6 d, 5 days of salt stress followed by 1 day on normal substrate. Actin was used as an internal control. Values represent mean ± standard deviation of three biological replicates. *P*-values were calculated using Student’s *t*-test and *P < 0.05 and **P < 0.01 and **P < 0.001 compared with control and 0 h, respectively.

To clarify the abundance of *GmWRKY45* transcripts in specific tissues, the total RNA were extracted from roots, stems, leaves, flowers, pods and seeds of soybean plants at first trifoliate (V1), full bloom (R2), and full seed (R6) stages, respectively. RT–qPCR analysis showed that *GmWRKY45* was expressed in all tissues examined and relative abundance of *GmWRKY45* transcripts in roots tissues (R2 and R6), flowers (R2) and pods (R6) (**Figure 2A**). In addition, we found that *GmWRKY45* transcripts in roots have an increasing response with growth advance (V1 to R6). The expression pattern of the leaves changed significantly, with lowest transcription observed at the V1, highest at the R2, and intermediate at the R6 stages. In stem tissues of R2 and R6 stages, the expression of *GmWRKY45* did not change significantly and was relatively stable.

The expression patterns of *GmWRKY45* under low Pi (2.5 µM KH_2_PO_4_) condition were evaluated by RT-qPCR using RNA samples extracted from leaves and roots, and normal Pi (1 mM KH_2_PO_4_) condition was used as control. The expression level of *GmWRKY45* increased significantly with time under low Pi treatment (**Figure 2B**). Specifically, the increases of *GmWRKY45* transcripts in the leaves and roots peaked at the 5th day and 10th day of Pi starvation respectively, and decreased rapidly after recovery with Pi for 1 day (11d/Recovery). In addition, we found that the intensity of *GmWRKY45* induced by low phosphorus in roots was higher than that in leaves (**Figure 2B**). This may imply that the response in *GmWRKY45* to low phosphorus is more sensitive in the root. To determine whether the up-regulation of *GmWRKY45* was specific to phosphate starvation, the responsiveness of *GmWRKY45* expression to salt stress was also investigated and we used normal condition as a control. Unlike phosphate starvation, *GmWRKY45* responded faster to salt exposure, although the induced expression declined gradually after the initial burst (**Figure 2C**). Taken together, these results suggest that *GmWRKY45* may play an important role in soybean growth and development and it is not only induced by phosphate starvation but also induced by salt stress.

### Overexpression of GmWRKY45 Affects the Adaptation of Transgenic Arabidopsis to Phosphate Starvation

**Figure 3:**
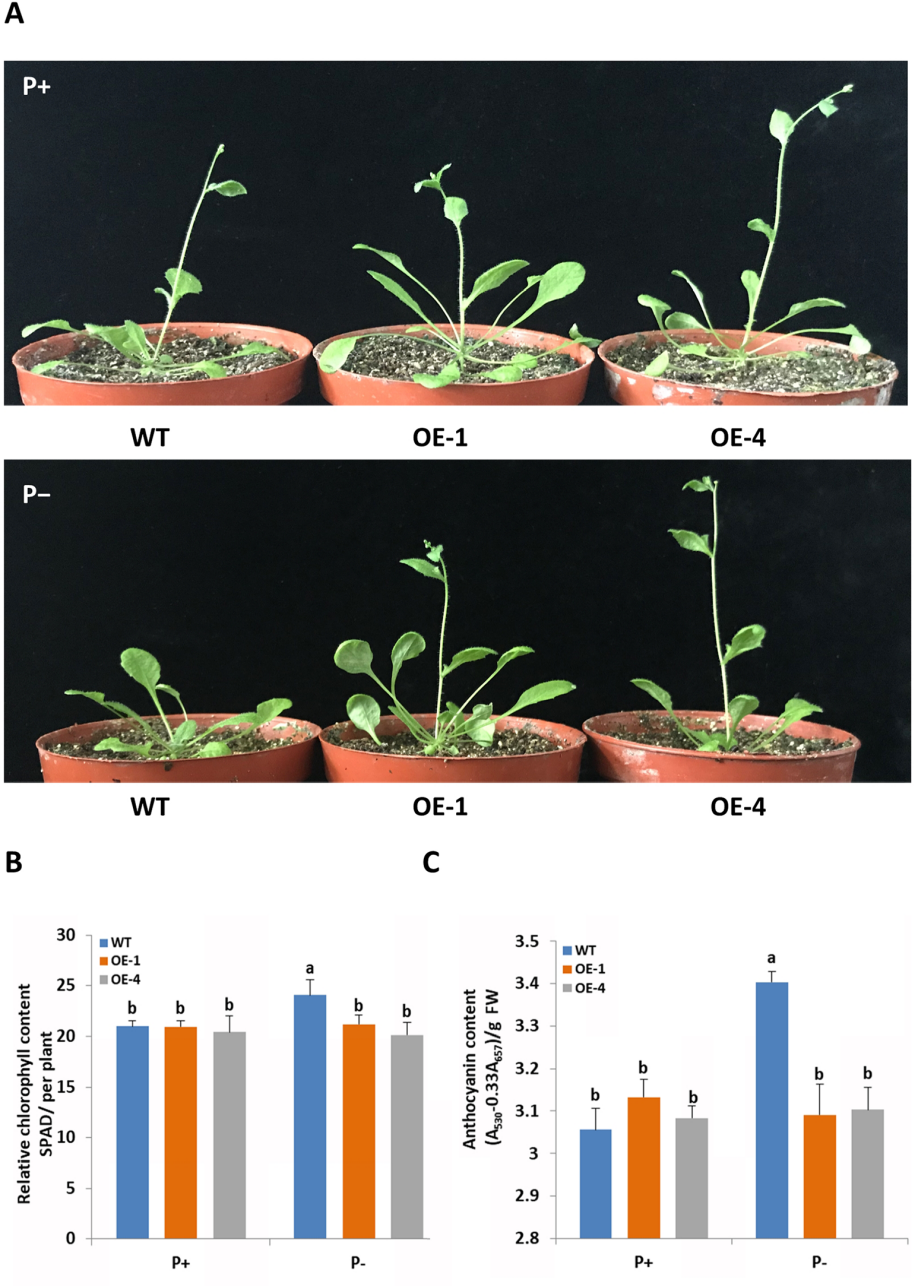
*GmWRKY45* enhances tolerance to phosphate starvation in *Arabidopsis*. **(A)** Two-week-old seedlings were grown in the greenhouse for 30 d under P+ (1 mM Pi) or P- (0.25 mM Pi) conditions. Comparison of chlorophyll **(B)** and anthocyanin **(C)** contents of WT and transgenic *Arabidopsis* plants (OE-1 and OE-4) in plants grown in the greenhouse for 30 d under P+ and P- conditions. Data are means of three replicates with errors bars indicating SD. Means with different letters are significantly different (one-way ANOVA, Duncan, *P* ≤ 0.05). The scale bars in the panels represent 1 cm.

To functionally characterize the role of *GmWRKY45* in response and adaptation to phosphate starvation, we constructed *GmWRKY45*-overexpressing transgenic *Arabidopsis* (**Supplemental Figure S1**). Two-week-old plants of transgenic *Arabidopsis* lines and the wild type *Arabidopsis* (WT) were exposed to a vermiculite medium containing Pi-sufficient (P+, 1 mM KH_2_PO_4_) and Pi-deficient (P−, 0.25 mM KH_2_PO_4_) conditions 30 d. Under P+ condition, we found the growth period of all plants was basically the same, with all plants at the stage of stem elongation (**Figure 3A** and **Supplemental Figure S2**). Under the P− condition, the growth period of *GmWRKY45*-overexpressing transgenic *Arabidopsis* lines was not affected, while the stem elongation of WT plants was inhibited (**Figure 3A** and **Supplemental Figure S2**).

In order to further verify whether *GmWRKY45* affects the adaptation of transgenic *Arabidopsis* to low phosphorus environment, we measured the chlorophyll and anthocyanin contents of *GmWRKY45*-overexpressing transgenic *Arabidopsis* and WT respectively. After P+ conditions 30 d, no significant differences in chlorophyll and anthocyanin contents were observed between WT and transgenic plants (**Figures 3B, C**). After P− condition 30 d, significant accumulation of chlorophyll and anthocyanin was observed in the WT. However, the contents of chlorophyll and anthocyanin in the *GmWRKY45*-overexpressing transgenic *Arabidopsis* lines under P− condition did not increase (**Figures 3B, C**). Previous studies have shown that plants produce more chlorophyll and anthocyanin under phosphorus deficiency stress (Marschner, 2011). These results mean that the low-phosphorus environment we established has a significant physiological stress effect on the WT, while the transgenic *Arabidopsis* is not affected. Therefore, we can determine that the overexpression of *GmWRKY45* attenuates the sensitivity of *Arabidopsis* to phosphate starvation and improves the adaptability of *Arabidopsis* to phosphate starvation.

### GmWRKY45 Enhances Tolerance to Phosphate Starvation May Partly Relate to Changes in Root System Architecture

**Figure 4:**
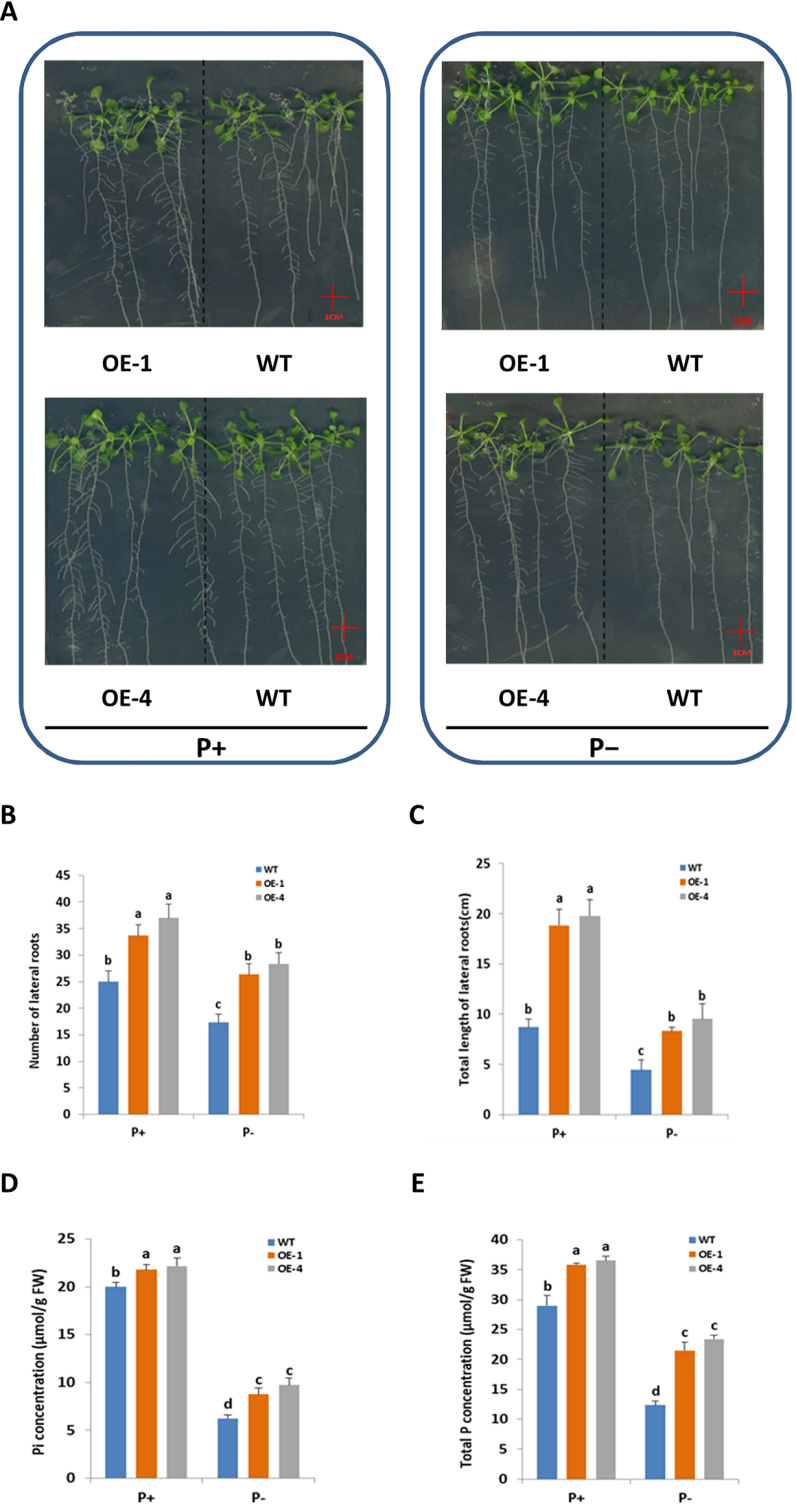
Overexpression of *GmWRKY45* increased lateral roots growth and phosphorus content in transgenic *Arabidopsis*. **(A)** WT and *GmWRKY45*-overexpressing transgenic *Arabidopsis* were grown under P+ and P- conditions for 15 days on vertically oriented petri plates. Total number of lateral roots per plant **(B)** and total length of lateral roots per plant **(C)** was determined in WT and transgenic *Arabidopsis* plants grown with P+ and P- on the 15 day. Inorganic phosphorus **(D)** and total phosphorus **(E)** contents were determined in WT and transgenic *Arabidopsis* plants grown with P+ and P- on the 15 day. Values are mean ± SE (n = 3), and different letters above the bars indicate that the means are statistically different (one-way ANOVA, Duncan, *P* ≤ 0.05).

Root system architecture (RSA) was one of the adaptive responses of plants to Pi status (López-Bucio et al., 2003), so it was logical to investigate *GmWRKY45* effect on RSA of *Arabidopsis*. To test whether the better growth of transgenic *Arabidopsis* in phosphate starvation was related to the change of RSA, the number and length of lateral roots of WT and transgenic plants grown in vertically oriented agar plates under P+ and P− conditions for 15 days were monitored (**Figure 4A**). In our experiment, deprivation of Pi significantly reduced lateral root growth of both WT and transgenic plants (*P* ≤ 0.05) (**Figures 4A–C**). However, compared with WT, both the number of lateral roots (**Figure 4B**) and the total length (**Figure 4C**) of transgenic plants were significantly increased (*P* ≤ 0.05) under P− condition. Lateral root (LR) plays an active role in plant phosphorus deficiency (Jia et al., 2018), so the increase of LR of transgenic *Arabidopsis* may be one of the reasons why transgenic plants grow faster and better than WT under P− condition. In addition, the LR of transgenic *Arabidopsis* is still more developed than WT under P+ condition (**Figures 4A–C**). This suggested that role of *GmWRKY45* in promoting LR development may be independent of plant Pi status. In this study, we did not detect significant differences in primary root (data not shown).

Furthermore, we measured the fresh and dry weight of the plants. The *GmWRKY45*-overexpressing transgenic *Arabidopsis* exhibited better growth than WT plants, as evidenced by the greater fresh weight, dry weight both under P+ and P- conditions (**Table 1**). The inorganic phosphorus and total phosphorus concentration of the WT and transgenic *Arabidopsis* were also investigated. We found that the concentration of inorganic phosphorus and total phosphorus in transgenic *Arabidopsis* was significantly higher than WT under both P+ and P- conditions (**Figures 4D, E**). These data proved that the regulation effect of *GmWRKY45* on lateral roots not only made the transgenic *Arabidopsis* better adapt to P- condition, but also performed better than the WT under P+ condition.

**Table 1:** Comparative values of fresh and dry weight of plants at 15 days under P+ and P- conditions, and the values are mean ± SD of three independent experiments with 5 seedlings being used in each experiment (*P*-values were calculated using Student’s *t*-test and ***P < 0.001 compared with WT).

Genotype	Fresh weight (mg)	Dry weight (mg)
P+(1mM Pi)		
WT	34.44 ± 3.67	2.46 ± 0.25
OE-1	58.24 ± 2.82***	4.3 ± 0.65***
OE-4	60.66 ± 2.76***	4.36 ± 0.68***
P-(0.25mM Pi)		
WT	23.12 ± 1.86	1.88 ± 0.16
OE-1	39.72 ± 1.79***	3.42 ± 0.13***
OE-4	40.16 ± 2.32***	3.46 ± 0.23***

### GmWRKY45 Regulates the Expression of Phosphate-Responsive Genes in Transgenic *Arabidopsis*

**Figure 5:**
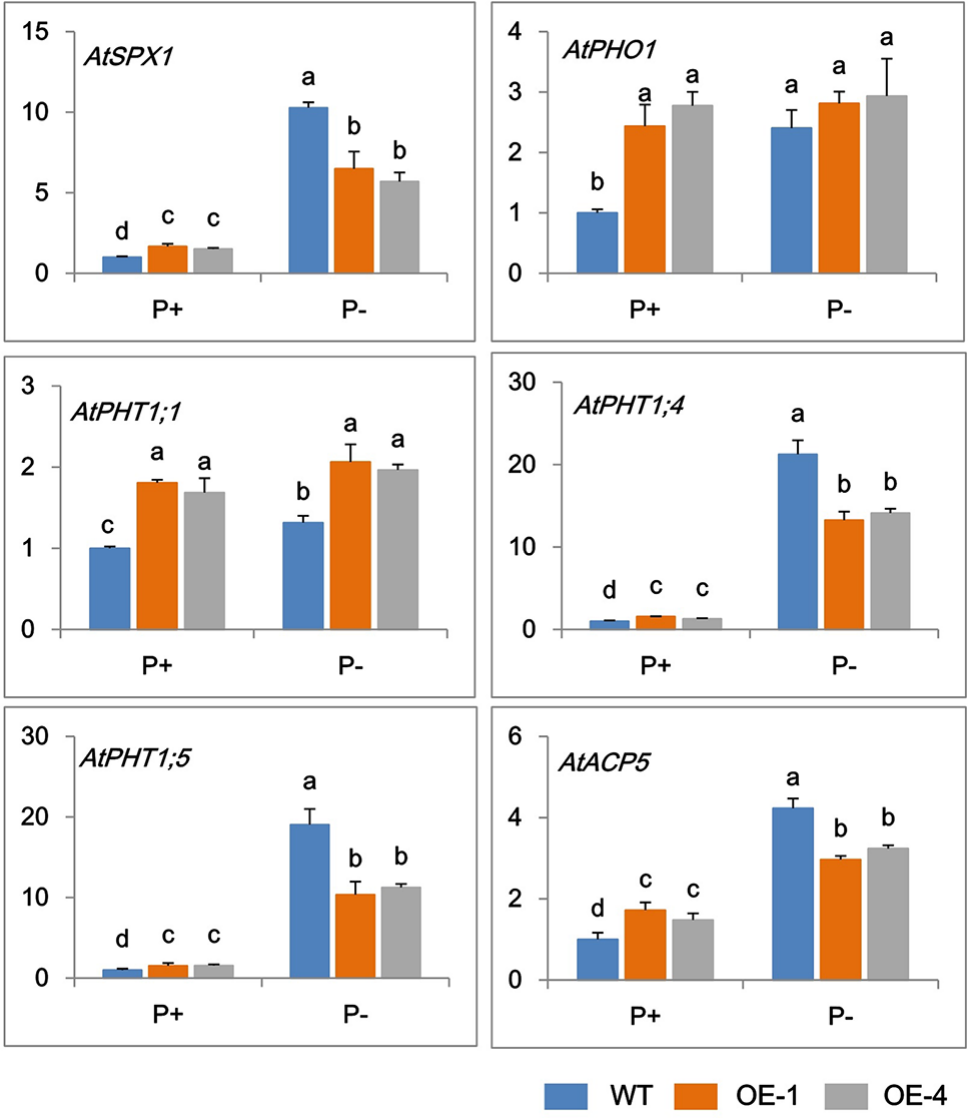
Expression change of phosphate-responsive genes in WT and *GmWRKY45*-overexpressing transgenic *Arabidopsis* plants. Total RNA samples were extracted from plants of seedling was done after growing them for 15 days on medium with P+ and P-. Expression was normalized to that of Actin. Data are means ± SD (n = 3). Means with different letters are significantly different (one-way ANOVA, Duncan, *P* ≤ 0.05).

In order to further clarify the mechanisms of *GmWRKY45* regulating the phosphate starvation response of transgenic *Arabidopsis*, the expression changes of several phosphate-responsive genes were monitored by RT-qPCR. Under P+ condition, the transcript abundance of *AtSPX1*, *AtPHO1*, *AtPHT1;1*, *AtPHT1;4*, *AtPHT1;5*, and *AtACP5* significantly increased in transgenic *Arabidopsis* compared with the WT (**Figure 5**). The steady-state transcript levels of all the tested genes increased in WT under P− condition (**Figure 5**), which is consistent with previous research (Del Pozo et al., 1999; Shin et al., 2004; Wang et al., 2004; Nagarajan et al., 2011; Puga et al., 2014). However, this increase in transcription levels was not significant in transgenic *Arabidopsis* lines. Under P- conditions, the transcript levels of *AtPHO1, AtPHT1;1*, and *AtACP5* did not increase in transgenic *Arabidopsis*, and the transcription levels of *AtSPX1, AtPHT1;4*, and *AtPHT1;5* were observed increased, but at a reduced level than that observed in WT (**Figure 5**). These results indicated that *GmWRKY45* enhanced the expression of these phosphate-responsive genes in transgenic *Arabidopsis* under normal condition, but changed the response of these phosphate-responsive genes to phosphate starvation.

### Overexpression of GmWRKY45 Enhances Salt Tolerance in Transgenic Arabidopsis

**Figure 6:**
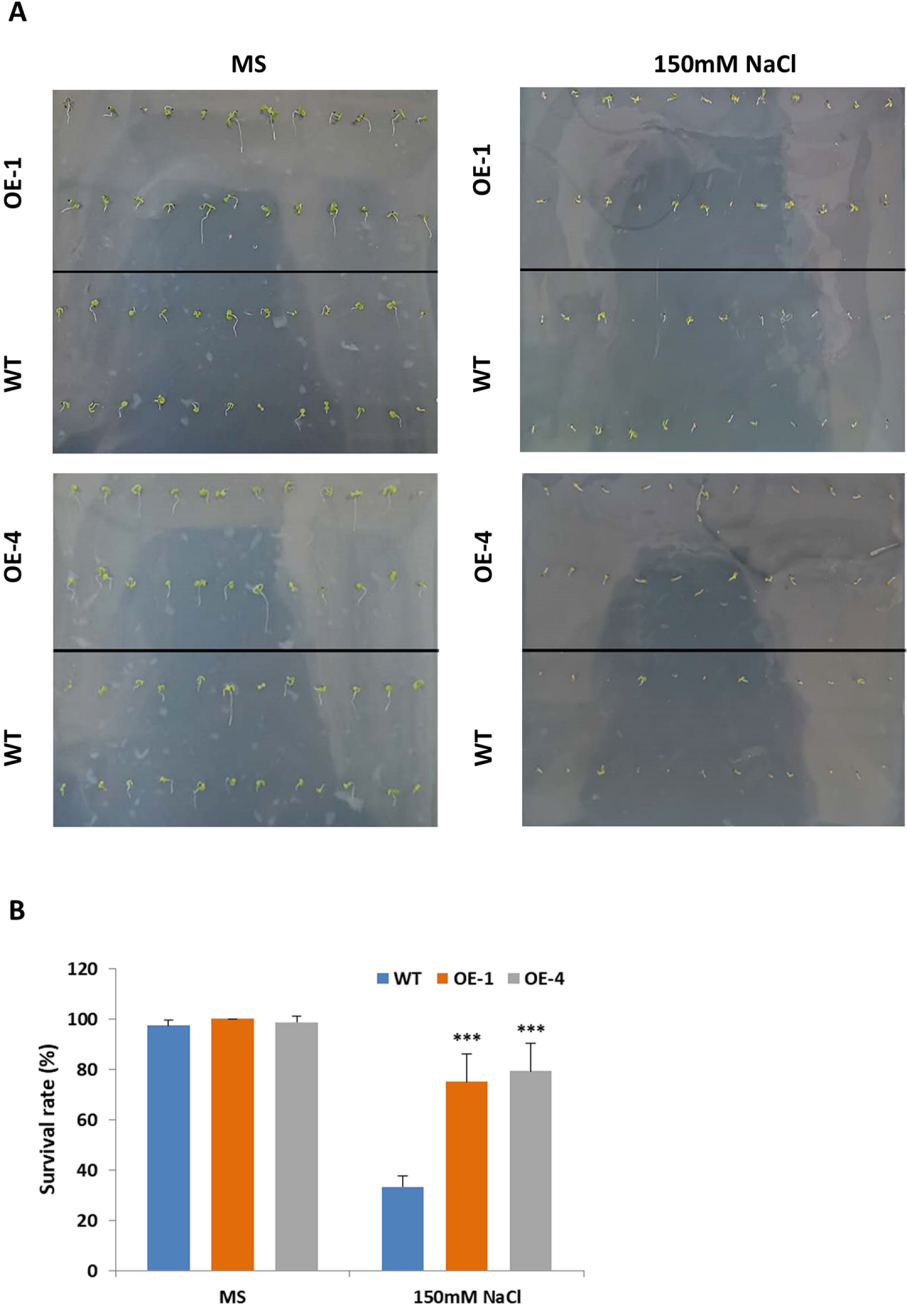
Survival rate of *Arabidopsis* seeds under different NaCl concentration treatments. **(A)** Survival situation of WT and *GmWRKY45*-overexpressing transgenic *Arabidopsis* seeds under 0 and 150 mM NaCl treatments. Photographs were taken at the end of experiments, after seven days. **(B)** Survival rate differences between WT and transgenic *Arabidopsis* seeds under 0 and 150 mM NaCl treatments, after 7 days. *P*-values were calculated using Student’s *t*-test and ***P < 0.001 compared with WT. Error bars represent SE (n = 3).

Salt stress had negative effects on plant growth and development by reducing survival rate, root length and fresh weight (Yeo, 1998). Studies have shown that salt-responsive TFs deal with high concentrations of salt by maintaining high survival rates and increasing their roots’ length (Hichri et al., 2017; Li et al., 2017). The results above told us that *GmWRKY45* induced by salt stress (**Figure 2C**) and overexpression of *GmWRKY45* increased the number and length of lateral roots of transgenic *Arabidopsis* (**Figure 4C**), so we speculated that overexpression of *GmWRKY45* might affected the salt tolerance in transgenic *Arabidopsis*. To test our speculation, WT and transgenic *Arabidopsis* seeds were cultured on MS medium with 0 and 150 mM NaCl for seven days. In the absence of salt stress, WT and transgenic *Arabidopsis* seeds showed non-significant survival rate differences (**Figures 6A, B**). In 150mM NaCl medium, the survival rate of transgenic lines was significantly higher than that of WT (*P* < 0.001) (**Figures 6A, B**). The experiments were repeated for three times and the results were consistent. One set of the experiments was shown.

**Figure 7:**
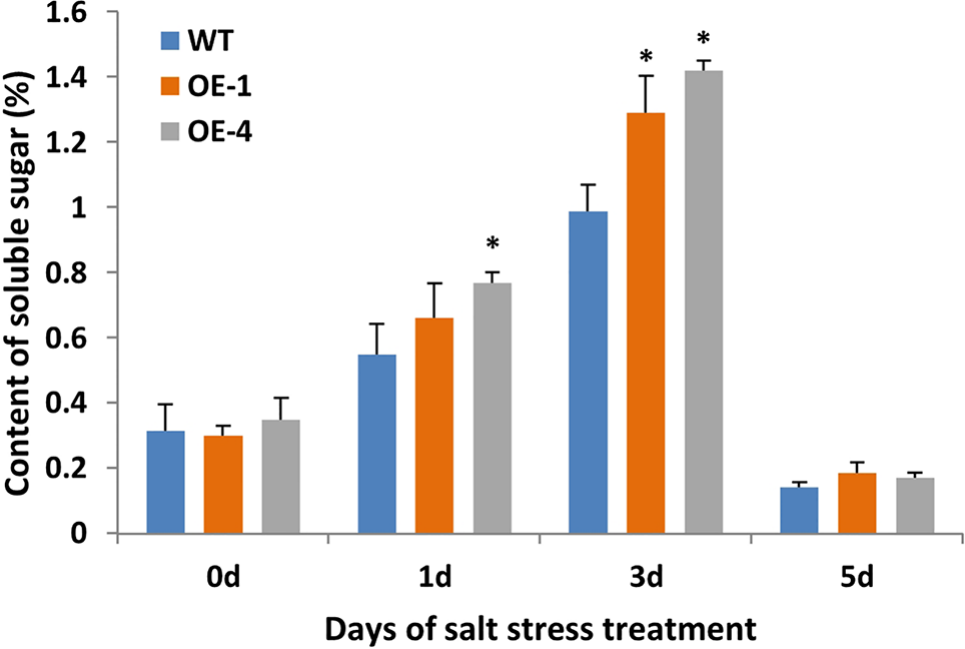
Soluble sugar content of WT and *GmWRKY45*-overexpressing transgenic *Arabidopsis* plants. *P*-values were calculated using Student’s *t*-test and *P < 0.05 compared with WT. Error bars represent SE (n = 3).

Plant cells with more soluble sugar accumulation had better tolerance to salt stress (Santa-Cruz et al., 1999). In order to evaluate the physiological changes of transgenic plants under salt stress, the content of soluble sugar as osmotic regulators in WT and transgenic *Arabidopsis* were measured. Transgenic *Arabidopsis* seedlings of two-week-old were treated with 150 mM NaCl for 5 days and WT seedlings were used as the control. After salt stress, seedlings of *GmWRKY45*-overexpressing transgenic *Arabidopsis* showed similar growth compared to WT seedlings (data not shown), but the transgenic *Arabidopsis* showed higher levels of soluble sugars compared to the WT (**Figure 7**). These results indicate that *GmWRKY45* may function in plant survival mechanism under high salinity levels and its salt resistance may be controlled by increasing soluble sugar in plants. Specifically, the soluble sugar content of WT and transgenic *Arabidopsis* both decreased rapidly on the 5th day of salt stress (**Figure 7**). We analyzed that it might be the continuous salt stress that caused the abnormal growth of plants.

### Overexpression of GmWRKY45 Changes Fertility of Transgenic Arabidopsis

**Figure 8:**
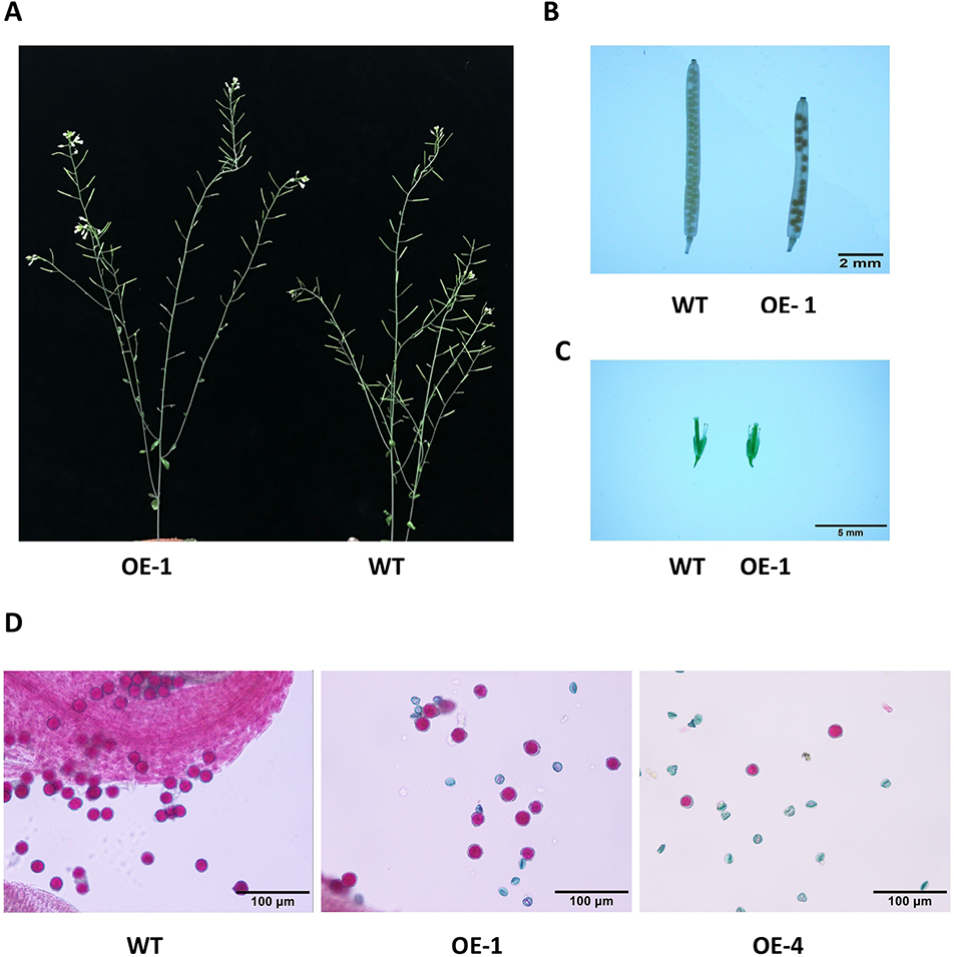
Overexpression of *GmWRKY45* in transgenic *Arabidopsis* changes its fertility. **(A)** Transgenic *Arabidopsis* and WT aboveground phenotypes. **(B)** The tenth silique (bottom to top) of transgenic *Arabidopsis* and WT. **(C)** Flower bud phenotype of transgenic *Arabidopsis* and WT. **(D)** Results of Alexander staining of pollens under magnification. Fertile pollens showed purplish red.

Many WRKY genes have been reported to affect plant development and growth throughout the entire development and growth period (Jiang and Yu, 2009; Zhang et al., 2010; Yang et al., 2016). In order to study the effect of *GmWRKY45* on the growth and development of *Arabidopsis*, we tested the growth phenotypes of WT and *GmWRKY45*-overexpressing transgenic *Arabidopsis*. At the early stage of plant growth, we did not find significant differences between transgenic *Arabidopsis* and WT. Interestingly, we found that the silique of the transgenic *Arabidopsis* was significantly shorter than WT at podding stage (**Figure 8A**), so we speculated that the overexpression of *GmWRKY45* might affect the fertility of the transgenic *Arabidopsis*. We selected the 1^st^, 5^th^, 10^th^, and 15^th^ silique from the bottom to the top, to measure the length and number of seeds per silique. At the same time, we decolorized them with alcohol and further observation under the microscope. We found that the silique of transgenic *Arabidopsis* was not only significantly shorter than WT, but also the number of seeds in the silique of transgenic *Arabidopsis* was also significantly less than WT (**Figure 8B** **and** **Table 2**). All transgenic lines had the same phenotype (**Supplemental Figure S3**). Here, we can determined that the fertility of transgenic *Arabidopsis* was affected. However, no differences were observed between flowers of transgenic *Arabidopsis* and WT (**Figure 8C**). Previous studies have shown that the development of male gametes in *Arabidopsis* requires a pollen specific transcription factor *WRKY34* and its homologous gene *WRKY2* (Lei et al., 2017). In view of the pollen development was closely related to plant fertility, we tried to carry out Alexander staining on the pollens of transgenic plants and WT. We found that nearly half of the pollens of transgenic plants were sterile which indicated that the fertility change of transgenic *Arabidopsis* could be caused by pollen abortion (**Figure 8D**).

**Table 2:** Plant silique length and number of seeds of WT and transgenic plants at podding stage (1^st^ S/P was representing the first silique from the bottom to up, other and so on), the values were mean ± SD of three independent experiments with four siliques were used in each experiment (*P*-values were calculated using Student’s *t*-test and ***P < 0.001 compared with WT).

Materials	Silique length (mm)	Seed number (per silique)
WT		
1^st^ S/P	9.78 ± 0.60	23.5 ± 1.73
5^th^ S/P	14.13 ± 0.69	45.75 ± 2.75
10^th^ S/P	14.52 ± 0.63	51.5 ± 1.29
15^th^ S/P	14.58 ± 0.36	55.5 ± 2.38
OE-1		
1^st^ S/P	8.92 ± 0.50	11.25 ± 1.50***
5^th^ S/P	8.32 ± 0.53***	14.75 ± 0.95***
10^th^ S/P	9.70 ± 0.66***	18.25 ± 1.50***
15^th^ S/P	8.84 ± 0.27***	11.50 ± 0.57***
OE-4		
1^st^ S/P	8.68 ± 0.34	11.00 ± 1.82***
5^th^ S/P	8.03 ± 0.65***	15.25 ± 0.95***
10^th^ S/P	9.26 ± 0.57***	18.50 ± 1.29***
15^th^ S/P	8.75 ± 0.42***	12.25 ± 0.95***

**Figure 9:**
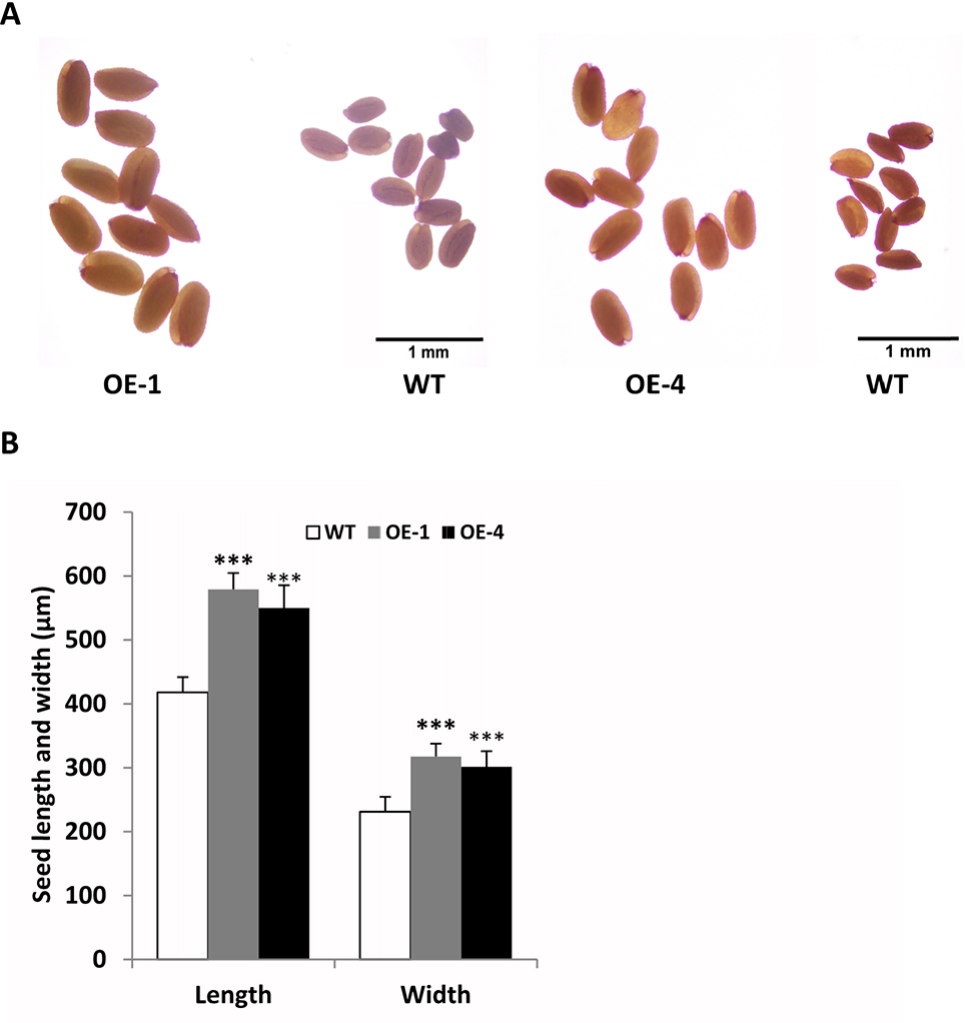
Overexpression of *GmWRKY45* in transgenic *Arabidopsis* changes its seed size. **(A)** Transgenic *Arabidopsis* compared with the WT on seed size comparison **(B)** Width and length of seeds from the transgenic *Arabidopsis* and WT. Data significantly different from the WT are indicated (***P < 0.001, Student’s *t*-test). Error bars represent SE (n = 3).

In addition, we found that transgenic seeds looked larger than WT seeds in **Figure 8B**, so we further observed and compared the seeds size of *GmWRKY45*-overexpressing transgenic *Arabidopsis* and WT. We randomly selected transgenic lines and WT seeds for further observation under the microscope, and we found that the transgenic seeds were indeed much larger (**Figure 9A**). Further, we determined seed length and width, and we found that the seeds of transgenic *Arabidopsis* were significantly longer and wider than the WT (**Figure 9B**). On the other hand, we measured the total yield per plant. We found that although the fertility of transgenic plants decreased, there was no difference in total yield between transgenic lines and WT (**Supplemental Figure S4**). These results confirm that the seeds of transgenic *Arabidopsis* become larger.

## Discussion

WRKY transcription factors play multiple roles in regulating plant developmental processes and responses to environmental challenges (Rushton et al., 2010), so in recent years, scientists have taken a keen interest in it. However, most functional analyses of WRKY transcription factors have been restricted to *A. thaliana* and *O. sativa*, and few WRKY family members from soybean have been reported relatively. In this study, a WRKY family gene was found in soybean by homology comparison, named *GmWRKY45*. *GmWRKY45* and *AtWRKY45* share similar structures and high sequence homology according to multiple comparisons and phylogenetic relationship analysis (**Figures 1A, B**). In the subcellular localization test, we found that *GmWRKY45* was localized in the nucleus (**Figure 1C**). Thus, *GmWRKY45* was a member of the soybean WRKY TFs family, and might function in the nucleus. RT–qPCR showed that the expression of *GmWRKY45* not only was induced by phosphate starvation, but was also induced by salt stress (**Figures 2B, C**). These facts suggest that *GmWRKY45* expression in soybean is not just related to one abiotic stress, like many other WRKY transcription factors such as *OsWRKY74*, not only was induced by deficiency of Pi and Fe, but also inhibited by N deficiency and cold stress (Dai et al., 2015). *GmWRKY45* was expressed in all tissues of soybean plants and strongly expressed in roots, flowers and pods tissues which may hint at its function (**Figure 2A**). Furthermore, we built the *GmWRKY45* pro::GUS transgenic *Arabidopsis* lines to further reveal the tissue expression pattern of *GmWRKY45*. Unfortunately, *GmWRKY45* pro::GUS activity was not detected in *GmWRKY45* promoter *Arabidopsis* at all growth stages (data not shown), indicating that its promoter maybe species-specific or the 2-kb region used in this experiment may not be sufficient.

In order to further study the functions of *GmWRKY45* in plants, we transformed the gene into *Arabidopsis* due to the difficulty of soybean transformation. *Arabidopsis* has been used in transgenic research for stress-tolerant genes from crops that are not easy for gene transformation analysis including soybean and wheat (Hao et al., 2011; He et al., 2011). We found that overexpression of *GmWRKY45* improved tolerance to phosphate starvation in transgenic *Arabidopsis* plants as under P- condition its growth was not inhibited relative to the WT (**Figure 3A** and **Supplemental Figure S2**). Through further physiological measurements, we found that the content of chlorophyll and anthocyanin did not increase in transgenic *Arabidopsis* in the low-phosphorus environment, while that in the WT increased significantly (**Figures 3B, C**). That’s not surprising. Lack of P causes plant cells to grow slowly, so the levels of chlorophyll are relatively high. Slow cell growth is also beneficial to anthocyanin formation (Devaiah et al., 2007; Marschner, 2011). Although *GmWRKY45* improved tolerance to phosphate starvation similarly as *AtWRKY45* in transgenic *Arabidopsis* plants, they may use different approaches. *AtWRKY45* is involved in *Arabidopsis* response to phosphate starvation by direct up-regulation of *AtPHT1;1* expression (Wang et al., 2014). In our study, we found the transgenic *Arabidopsis* has better adaptability to phosphate starvation, which may be partly due to overexpression of *GmWRKY45* improving lateral root development in transgenic *Arabidopsis* (**Figure 4** and **Table 1**). Lateral root initiation and elongation may play an important role in the uptake of immobile nutrients such as phosphorus by increasing soil exploration and phosphorus acquisition (Zhu et al., 2005). In addition, we found that transgenic *Arabidopsis* had higher survival rate (**Figure 6**) and content of soluble sugar (**Figure 7**) compared with WT under salt stress. Overexpression of *GmWRKY45* results in partial abortion of the pollens, shorter silique and fewer seeds per pod in *Arabidopsis* (**Figure 8**, **Table 2**, and **Supplemental Figure S3**). However, although the number of seeds decreased, the yield of seed per plant did not decrease, which might be due to the transgenic *Arabidopsis* seed size larger than WT (**Figure 9** and **Supplemental Figure S4**). Studies have shown that the expression difference of *SoyWRKY15a* can affect soybean seed size (Gu et al., 2017), and *GmWRKY45* may be another WRKY protein related to seed size regulation in soybean. These results show that *GmWRKY45* has a complex regulatory network in plants and is closely related to plant stress resistance and development.

Some phosphate-responsive genes have been shown to play an important role in phosphate response in *Arabidopsis*. *AtSPX1* is one of Pi-dependent inhibitors of PHOSPHATE STARVATION RESPONSE 1 (PHR1) activity and is highly responsive to Pi starvation (Puga et al., 2014). *AtPHO1* has previously been shown to participate in the transport of Pi from roots to shoots, and the gene expression profile normally triggered by Pi deficiency is suppressed in plants with low *AtPHO1* expression (Rouached et al., 2011). *AtPHT1;1*, *AtPHT1;4*, and *AtPHT1;5* belong to members of the *Arabidopsis* Pht1 phosphate transporter family. *AtPHT1;1* and *AtPHT1;4* play a major role in phosphate acquisition from both low- and high-phosphate environments (Shin et al., 2004), AtPHT1;5 plays a critical role in mobilizing Pi from P source to sink organs in accordance with developmental cues and P status (Nagarajan et al., 2011). *AtACP5* is a type 5 acid phosphatase gene from *Arabidopsis* which is induced by phosphate starvation and other types of phosphate mobilising/oxidative stress conditions (Del Pozo et al., 1999). Our results showed the *GmWRKY45*-overexpressing transgenic *Arabidopsis* enhanced the expression of these phosphate-responsive genes under Pi-sufficient condition (**Figure 5**), which might be one of the reasons why transgenic *Arabidopsis* had highter P concentration than WT (**Figures 4D, E**). Under Pi-deficient condition, the expression of these phosphate-responsive genes was increased in *GmWRKY45*-overexpressing transgenic *Arabidopsis,* but the increase was not stronger than WT (**Figure 5**). This may mean that overexpression of *GmWRKY45* reduces the sensitivity of *Arabidopsis* to low phosphorus environment. The expression of several phosphate-responsive genes was reduced in low phosphorus insensitive mutants grown in low P media (Lenin et al., 2006).

Lateral root (LR) development plays an important role in many plant stress tolerances, not only in phosphate starvation. For example, excessive Fe has different effects on the development of LR in different parts of *Arabidopsis* root system, and the inhibition of LR activation initiation is only seen in roots newly form during excess Fe exposure (Kronzucker et al., 2015); Salt stresses causes an extended quiescent phase in postemergence LRs whereby the rate of growth is suppressed for several days and that is correlated with sustaining ABA response in LRs (Lina et al., 2013); Poplar *PtabZIP1*-like enhances LR formation and biomass growth under drought stress (Dash et al., 2017). In our study, we found that the lateral roots of *GmWRKY45-*overexpressing transgenic *Arabidopsis* to grow well even under stress-free condition (**Figure 4**), which means that transgenic *Arabidopsis* may also perform well in other adverse soil conditions. We used PlantCARE (http://bioinformatics.psb.ugent.be/webtools/plantcare/html/) to analyze the promoter of *GmWRKY45* and found that contains many environmental stress responses and plant hormone response cis elements (**Supplemental Table S5**). In this paper, only phosphate starvation and salt stress are examined, so further studies are needed to clarify *GmWRKY45* expression and the performance of the transgenic *Arabidopsis* plants in other adverse soil conditions. Furthermore, improved root systems and survival rate are considerable interest in agriculture; the pollen fertility and seed size profoundly influence total harvest. Thus, it is meaningful to study the underlying regulatory molecular mechanisms in greater detail.

## Conclusions

In summary, this study characterized *GmWRKY45*, a WRKY TFs belonging to group IIc of the WRKY protein family that is localized at the nucleus in soybean. The *GmWRKY45* protein acts as an important regulator of phosphate starvation responses, so that overexpression of *GmWRKY45* resulted in enhanced tolerance to phosphate starvation, more and longer lateral roots, and changed expression of phosphate-responsive genes in transgenic *Arabidopsis* plants. In addition, our results also showed that *GmWRKY45* enhanced tolerance to salt stress, and changed fertility in transgenic *Arabidopsis*. Further works on the role of *GmWRKY45* to other abiotic stress are under way in our laboratory. We are also working on the construction of soybean overexpression and mutant materials, with a view to further studying the regulatory mechanism of *GmWRKY45* on abiotic stress and development of soybean.

## Data Availability Statement

Publicly available datasets were analyzed in this study. This data can be found here: Glyma.03g220800.

## Author Contributions

CL and SY designed the experiments. CL, XL, HR, JZ, and FX finished the experiments. CL and XL analyzed the data. CL finished the manuscript. SY and JG revised the paper. All authors approved the paper.

## Funding

This work was supported by the National Transgene Science and Technology Major Program of China (2016ZX08004-005), the Fundamental Research Funds for the Central Universities (KYT201801) and the Program for Changjiang Scholars and Innovative Research Team in University (PCSIRT_17R55).

## Conflict of Interest

The authors declare that the research was conducted in the absence of any commercial or financial relationships that could be construed as a potential conflict of interest.
