# Supplementary material for: GmWRKY45 Enhances Tolerance to Phosphate Starvation and Salt Stress, and Changes Fertility in Transgenic Arabidopsis
Source: Front Plant Sci. 2020 Jan 29;10:1714. doi: 10.3389/fpls.2019.01714 (PMC7000756; doi:10.3389/fpls.2019.01714)
Supplement: Supplementary file 1 [file DataSheet_1.pdf]

Supplemental Figures

Supplemental Fig. S1

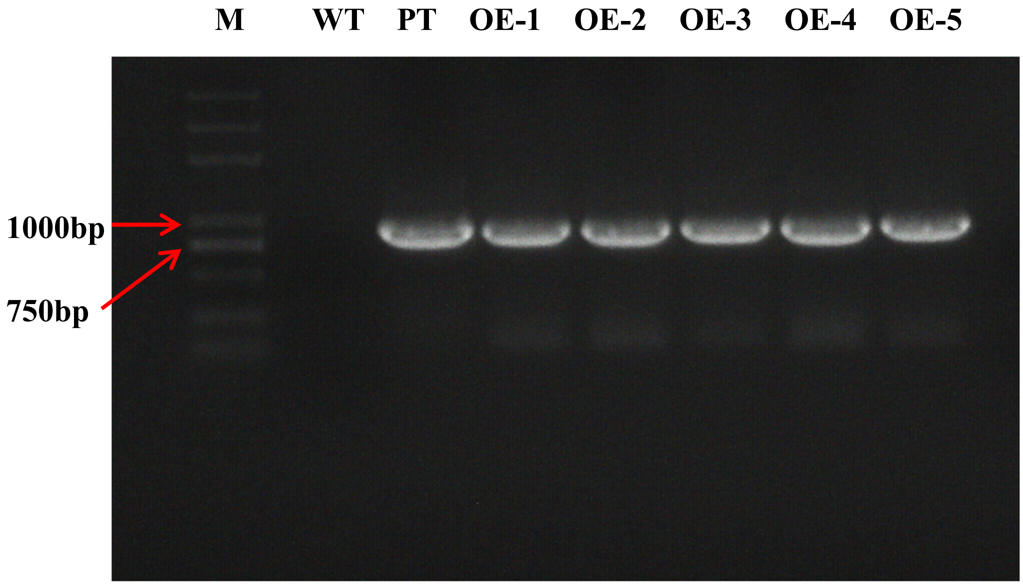

Supplemental Fig. S1. The *GmWRKY45*-overexpressing transgenic *Arabidopsis* were identified by PCR, and *GmWRKY45* gene had a size of 834bp fragment**.** M, DNA Marker DL 5000; WT, wild type *Arabidopsis* lines; PT, the binary vector pCAMBIA3301-*GmWRKY45* (positive control); OE-1 to 5, independent transgenic *Arabidopsis* lines.

Supplemental Fig. S2

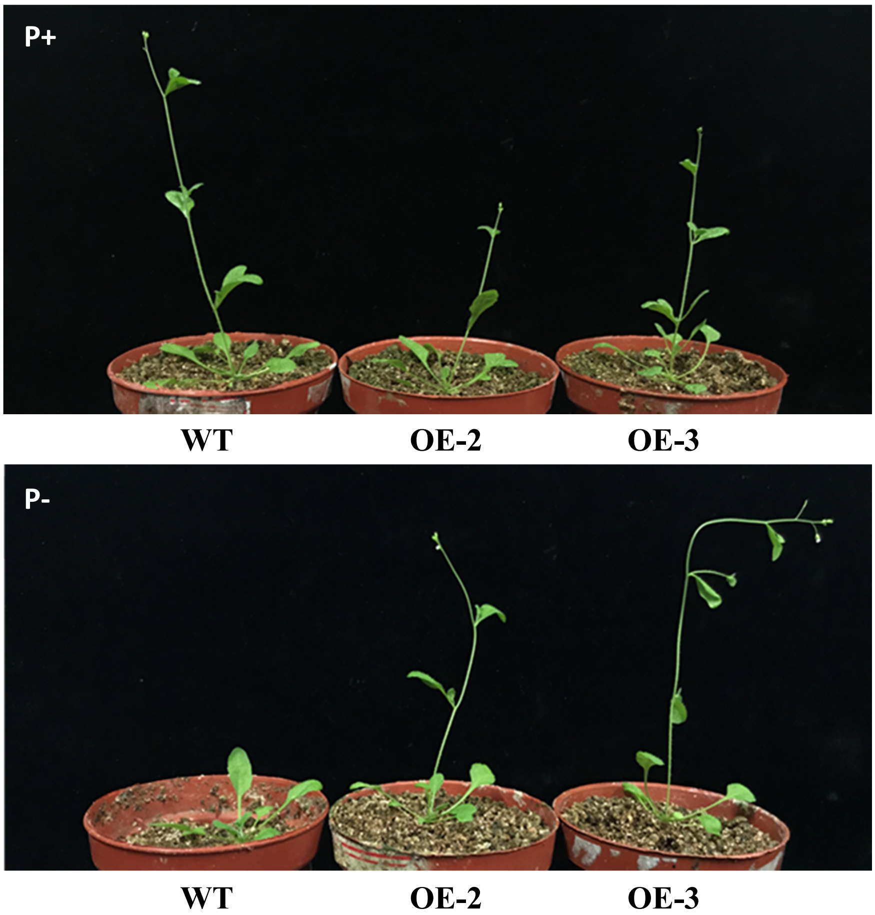

Supplemental Fig. S2. *GmWRKY45* enhanced tolerance to phosphate starvation in *Arabidopsis*. Tow-week-old seedlings were grown in the greenhouse for 30d under P+ (1 mM Pi) or P- (0.25 mM Pi) conditions.

Supplemental Fig. S3

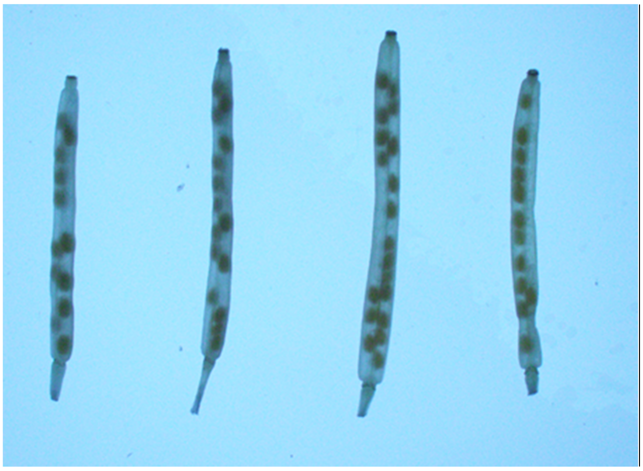

OE-2 **OE-3** **OE-1** **OE-4**

Supplemental Fig. S3. Silique from the 10th flower (bottom to top) formed within the primary inflorescences of *GmWRKY45*-overexpressing transgenic *Arabidopsis*.

Supplemental Fig. S4

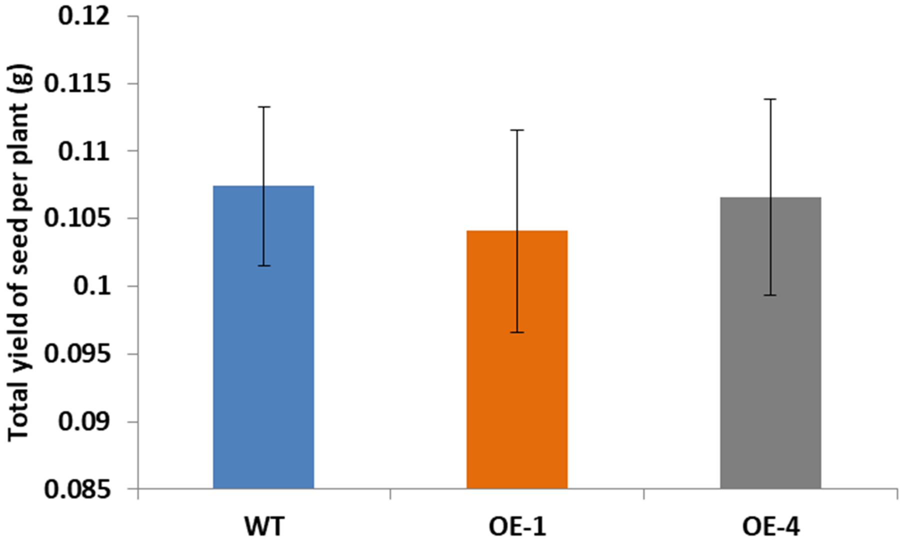

Supplemental Fig. S4. Total seed yield per plant of WT and *GmWRKY45*-overexpressing transgenic *Arabidopsis* (OE-1 and OE-4). Error bars represent SE (n = 3).

Supplemental Tables

Supplemental Table S1. A list of primers used for soybean qRT-PCR.

Gene	Forward primer （5’-3’）	Reverse primer （5’-3’）
GmWRKY45	GAAACGTGTGGAGCGATCTT	CCATAACTGGGCTTGGATGC
Actin	CCTCAACCCAAAGGTCAACAG	GACCAGCGAGATCCAAACGAA

Supplemental Table S2. A list of primers used for *Arabidopsis* qRT-PCR.

Gene	Forward primer （5’-3’）	Reverse primer （5’-3’）
ATSPX1	CCGTCGGAATATCGAAAGAA	TCCTTGACAAGCTTGAACAATAAG
ATPHO1	TGGTTCTCCGGAACAAGAAC	TGACTTCAAGTGACGCCAAG
ATPHT1;1	CCTTTGGGTTCCTATATGCG	ATTCATTTATTTGGATGGTTGTTCA
AtPHT1:4	TGGGATTCTTCACTGATGCTTACG	CGAAAATACCACCAGCCATGATTC
AtPHT1:5	CGGAGGAATCGTGTCTCTCATCGT	TGGAAGAGGTTGCTGCTGTAATAGG
AtACP5	GTGAGCTTCAGAGATTTATAGAGCC	TTTGACATAAGAGTTGCGAGATG
Actin2-8	ACGGTAACATTGTGCTCAGTGGTG	CTTGGAGATCCACATCTGCTGGA

Supplemental Table S3. A list of primers used for cDNA cloning

Gene	Forward primer （5’-3’）	Reverse primer （5’-3’）
GmWRKY45	AACACGGGGGACTCTTGACAATGGAGAATACTAAGATGATGG	GCCCTTGCTCACCATAGATCTATCTTCTTTCAACATATGTGAA

Supplemental Table S4. A list of primers used for promoter cloning

Gene	Forward primer （5’-3’）	Reverse primer （5’-3’）
GmWRKY45	GAGCTCGGTACCCGGGGATCCAAATTAAAACATTGTGTAGCCTCCC	TAGAAATTTACCCTCAGATCTATGATCTCTCTCAAATGCCCAAA

Supplemental Table S5. Putative cis-regulatory elements involved in the promoter of *GmWRKY45*

cis-regulatory elements	Motif	Number	Description
ABRE	ACGTG/TACGTGTC	5	cis-acting element involved in the abscisic acid responsiveness
ARE	AAACCA	1	cis-acting regulatory element essential for the anaerobic induction
Box 4	ATTAAT	2	part of a conserved DNA module involved in light responsiveness
CAAT-box	CAAAT	16	common cis-acting element in promoter and enhancer regions
G-box	TACGTG	2	cis-acting regulatory element involved in light responsiveness
GC-motif	CCCCCG	1	enhancer-like element involved in anoxic specific inducibility
GT1-motif	GGTTAA	1	light responsive element
Gap-box	CAAATGAA(A/G)A	1	part of a light responsive element
TATA-box	ATATAT	43	core promoter element around -30 of transcription start
TCA-element	CCATCTTTTT	1	cis-acting element involved in salicylic acid responsiveness
TCT-motif	TCT-motif	1	part of a light responsive element
W box	TTGACC	1	
